# Association of Intratumoral Microbiota With Prognosis in Patients With Nasopharyngeal Carcinoma From 2 Hospitals in China

**DOI:** 10.1001/jamaoncol.2022.2810

**Published:** 2022-07-14

**Authors:** Han Qiao, Xi-Rong Tan, Hui Li, Jun-Yan Li, Xiao-Zhong Chen, Ying-Qin Li, Wen-Fei Li, Ling-Long Tang, Guan-Qun Zhou, Yuan Zhang, Ye-Lin Liang, Qing-Mei He, Yin Zhao, Sheng-Yan Huang, Sha Gong, Qian Li, Ming-Liang Ye, Kai-Lin Chen, Ying Sun, Jun Ma, Na Liu

**Affiliations:** 1 Department of Experimental Research, State Key Laboratory of Oncology in South China, Sun Yat-sen University Cancer Center, Guangzhou, People’s Republic of China; 2 Collaborative Innovation Center of Cancer Medicine, Sun Yat-sen University Cancer Center, Guangzhou, People’s Republic of China; 3 Guangdong Key Laboratory of Nasopharyngeal Carcinoma Diagnosis and Therapy, Sun Yat-sen University Cancer Center, Guangzhou, People’s Republic of China; 4 Zhongshan School of Medicine, Sun Yat-sen University, Guangzhou, People’s Republic of China; 5 Department of Radiation Oncology, State Key Laboratory of Oncology in South China, Sun Yat-sen University Cancer Center, Guangzhou, People’s Republic of China; 6 Cancer Hospital of the University of Chinese Academy of Science (Zhejiang Cancer Hospital), Institute of Cancer and Basic Medicine (IBMC) Chinese Academy of Sciences, Hanzhou, People’s Republic of China

## Abstract

**Importance:**

Microbiota-tumor interactions have qualified microbiota as a promising prognostic biomarker in various types of cancers. Although the nasopharynx acts as a crucial niche of the upper respiratory tract microbiome, whether the intratumoral microbiota exists and its clinical significance in nasopharyngeal carcinoma (NPC) remain uncertain.

**Objective:**

To evaluate the clinical significance of intratumoral microbiota for individual prognostication in patients with NPC.

**Design, Setting, and Participants:**

This retrospective cohort study included NPC biopsy samples from 2 hospitals: Sun Yat-sen University Cancer Center (Guangzhou, China) and Zhejiang Cancer Hospital (Hangzhou, China) between January 2004 and November 2016, with follow-up through November 2020. A total of 802 patients were included according to the following criteria: with histologically proven NPC, without distant metastasis at initial diagnosis, had not received antitumor treatment before biopsy sampling, aged between 18 and 70 years, with complete medical records and regular follow-up, without a history of cancer, and successfully extracted enough DNA for experiments.

**Main Outcomes and Measures:**

The primary end point was disease-free survival, and the secondary end points included distant metastasis–free survival and overall survival. To assess the existence and load of intratumoral microbiota in 96 patients with NPC with or without tumor relapse, 16S rRNA sequencing and quantitative polymerase chain reaction were used. The associations between intratumoral bacterial load and clinical outcome were evaluated in 241 fresh-frozen NPC samples (training cohort) and validated in paraffin-embedded NPC samples of internal (n = 233) and external (n = 232) validation cohorts. Metagenomic and transcriptome analyses were performed to ascertain the origin and underlying mechanism of intratumoral bacteria.

**Results:**

A total of 802 patients with NPC (mean [SD] age, 46.2 [10.6] years; 594 [74.1%] male) were enrolled. Microbiota presented within NPC tumor tissues, among which *Corynebacterium* and *Staphylococcus* predominated. Patients with a high bacterial load in the training cohort had inferior rates of disease-free survival (hazard ratio [HR], 2.90; 95% CI, 1.72-4.90; *P* < .001), distant metastasis-free survival (HR, 3.18; 95% CI, 1.58-6.39; *P* < .001), and overall survival (HR, 3.41; 95% CI, 1.90-6.11, *P* < .001) than those with a low bacterial load, a finding that was validated by the internal and external validation cohorts. Single-nucleotide variant analysis revealed that the nasopharyngeal microbiota was the main origin of NPC intratumoral bacteria. Transcriptome and digital pathology analyses demonstrated that a higher intratumoral bacterial load was negatively associated with T-lymphocyte infiltration.

**Conclusions and Relevance:**

Intratumoral bacterial load was a robust prognostic tool for patients with NPC in this cohort study, indicating potential guidance for treatment decisions in patients at different levels of risk of malignant progression.

## Key Points

**Question:**

Is there characteristic microbiota in nasopharyngeal carcinoma (NPC) tissues and, if so, is it associated with prognosis?

**Findings:**

In this multicenter cohort study including 802 patients with NPC, we confirmed the existence of microbiota within NPC tissues, which mainly originated from the nasopharynx. Intratumoral bacterial load was associated with poor survival in patients with NPC and was negatively associated with T-lymphocyte infiltration.

**Meaning:**

The results suggest that the intratumoral bacterial load may be a reliable prognostic indicator for patients with NPC.

## Teaser

This multicenter cohort study analyzes the associations between intratumoral microbiota and prognostication in patients with nasopharyngeal carcinoma.

## Introduction

Currently, tumor node metastasis staging is widely used to provide prognostic information and guide treatment strategies for patients with nasopharyngeal carcinoma (NPC).^1,2,3^ About 30% of patients with the same stage who receive similar treatment regimens exhibit local recurrence or distant metastasis,^4^ suggesting that the anatomy-based staging system is insufficient for determining individualized therapy. Emerging interpretation of molecular variations in pathogenesis heightens the demand for molecular tools to stratify patients with NPC with respect to different prognoses.^5,6,7,8^ However, discerning novel efficient biomarkers remains imperative.

Microbiota is regarded as an invisible organ modulating numerous physiological functions, and dysbacteriosis has been implicated as a contributor to diseases covering various systems.^9^ In particular, microbiota aggressively participates in the tumorigenesis and progression of various cancers through inflammation-mediated immune suppression, metabolic pathways, and bacterial-derived toxins.^10,11^ Recently, gut microbiota has gained widespread attention owing to benefits found with probiotic-based fecal microbiota transplantation in improving prognoses in cancer patients.^12^ In addition, studies have reported that microbiota from the vagina, lung, and oral cavity are closely associated with relevant tumor occurrence and progression.^13,14,15^ Thus, microbiota-tumor interactions have qualified microbiota as a promising biomarker and therapeutic target for diverse tumors.

The revelation of microbiota within several tumors that were initially considered sterile reinforces the concept of intratumoral microbiome.^16^ Emerging evidence has confirmed the critical involvement of the intratumoral microbiota on oncogenic behaviors in pancreatic cancer, lung cancer, and breast cancer.^14,17,18^ Unlike gastrointestinal cancers, respiratory tract cancers represent tumors with a relatively low bacterial biomass, which makes the bacterial load a critical consideration involved in tumor initiation and progression, as elucidated in lung cancer.^19^ As a microbial risk factor, the association of NPC with Epstein-Barr virus has been firmly established.^20^ While the nasopharynx acts as a crucial niche of the upper respiratory tract microbiome, whether the intratumoral microbiota exists and its clinical implications in NPC remain largely unknown.

We conducted what is to our knowledge the first and largest cohort study to assess the existence of intratumoral bacteria and its clinical significance in patients with NPC. We also explored the origination of intratumoral bacteria and its underlying mechanism involved in NPC tumor relapse.

## Methods

### Study Population

**Figure 1.**
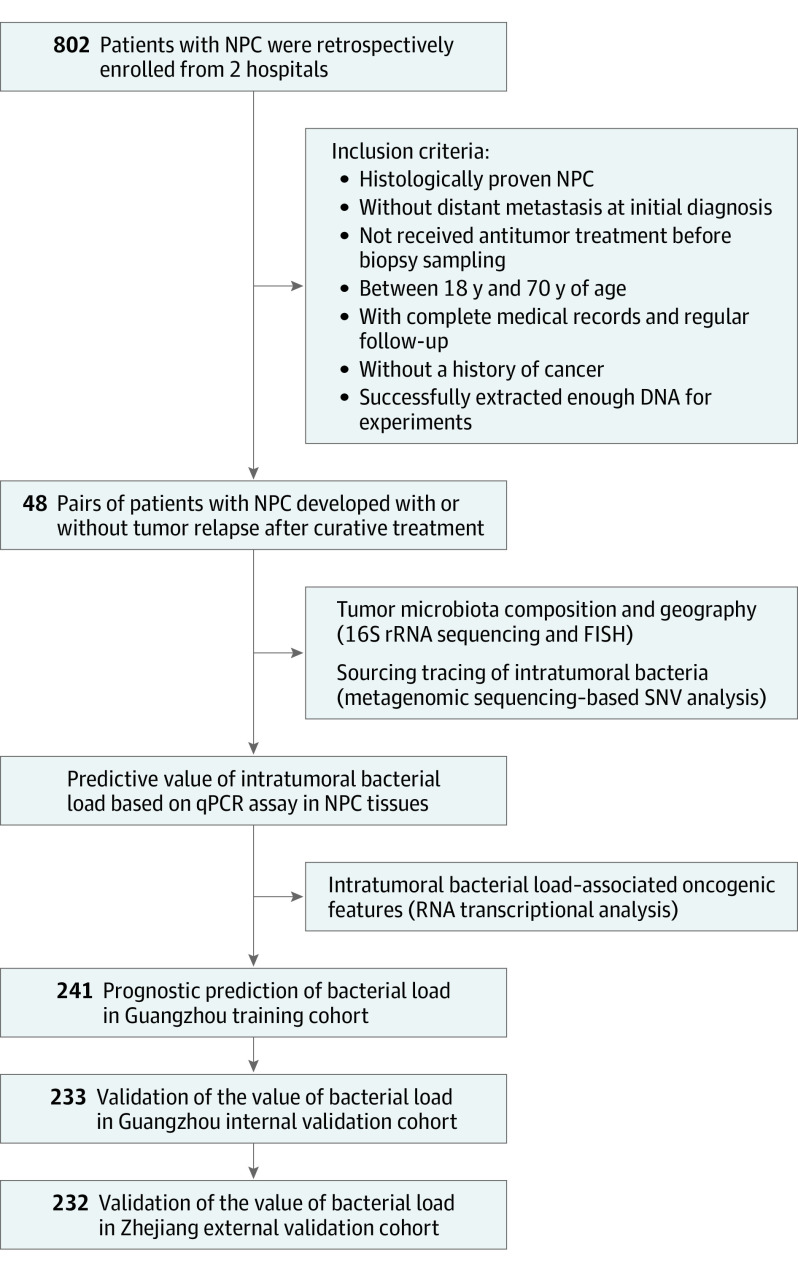
Study Design NPC indicates nasopharyngeal carcinoma; FISH, fluorescence in situ hybridization; qPCR, quantitative polymerase chain reaction; SNV, single-nucleotide variant.

We retrospectively acquired 802 pretreatment biopsy tissues from patients with nonmetastatic NPC using strict eligibility criteria (Figure 1). Of these, 570 samples were collected from Sun Yat-sen University Cancer Center (Guangzhou, China). Among them, 96 fresh-frozen tissues from 48 paired patients with NPC with relapse within 3 years or without relapse for more than 5 years were designated to the discovery cohort, 241 fresh-frozen tissues gathered between July 2010 and November 2016 were assigned as training cohort, and 233 paraffin-embedded tumors collected between January 2004 and April 2007 served as an internal validation cohort. An additional 232 paraffin-embedded tumors from Zhejiang Cancer Hospital (Zhejiang, China) between January 2004 and December 2005 were designated as an external validation cohort. All patients underwent radiotherapy, and 694 (86.5%) accepted platinum-based chemotherapy. No patients had disease progression while receiving treatments. All patients were restaged according to the 8th American Joint Committee on Cancer staging system,^21^ and any contradiction was settled by consensus.

We also prospectively collected biopsy tissues, nasopharyngeal swabs, saliva, and fecal samples from 20 patients with NPC who did not receive antibacterial treatment for 2 weeks before sampling. The institutional ethical review boards of both hospitals approved this study for analyzing anonymous data. The requirement of informed consent was waived for the retrospective analysis, and written informed consent was obtained from each patient for the prospective study. The study followed the Strengthening the Reporting of Observational Studies in Epidemiology (STROBE) reporting guideline.

### Laboratory Methods

The DNA and RNA were isolated by the AllPrep DNA/RNA Micro Kit, QIAamp DNA FFPE Tissue Kit, or QIAGEN DNeasy PowerSoil Kit (QIAGEN GmbH). Sterile pipettes, pipette tips, and nonenzymatic kit components were UV-irradiated for at least 1 hour prior to use.

Bacterial 16S rRNA V3-V4 region was amplified for library construction^22^ and sequenced on the Illumina Nova6000 platform at Magigene Biotechnology Co, Guangzhou, China. Eleven batch controls (DNA extraction, polymerase chain reaction, and sequencing platform) were included. Raw reads were processed by QIIME 2 version 2020.6 (Knight Lab and Caporaso Lab) to obtain amplicon sequence variants.^23^ Representative sequence sets were used for taxonomy classification with a naive Bayes classifier according to the SILVA version 132 (Ribocon GmbH) 16S database.^24^ A contamination-removal procedure (eFigure 1 and eMethods in Supplement 1) was established to filter contaminant amplicon sequence variants. Bacterial genera with amplicon sequence variant counts of 5 or higher were applied to produce a taxonomic tree by GraPhlAn version 1.1.3 (Huttenhower Lab).^25^ An even sampling depth of 5000 sequences per sample was used for diversity measurement (eFigure 2 in Supplement 1). Differential taxa between groups were analyzed by the DESeq2 package version 1.30.1 (Bioconductor).^26^

Sequential tumor sections were used for hematoxylin and eosin and fluorescence in situ hybridization assays by an Enhanced Sensitive ISH Detection Kit IV (BOSTER). A Cy3-labeled probe (EUB338-GCTGCCTCCCGTAGGAGT) targeting 16S rRNA was designed as previously reported.^16^ Immunohistochemistry was performed using an antibody against bacterial lipopolysaccharide (HycultBiotech). For intratumoral bacteria quantification, an equal amount of DNA (250 ng/μL) was used to amplify the V1-V2 region on the LightCycler 480 Real-Time PCR System (Roche) with specific primers (27F: AGAGTTTGATCMTGGCTCAG; 338R: TGCTGCCTCCCGTAGGAGT) as reported previously.^19^ We included DNA extraction and paraffin controls from each cohort. *Escherichia coli* genomic DNA was used to generate a standard curve (eFigure 3 in Supplement 1), which was used to quantify the absolute bacterial load by averaging 3 technical repeats.

Intratumoral bacteria were dipped with sterile swabs and cultivated at 37 °C for 48 hours in aerobic and anaerobic incubators. Environmental and swab controls were set up. Strains were cultivated, purified, and identified by MALDI-TOF mass spectrometry. All isolated strains and nasopharyngeal swab, saliva, and fecal samples from the same patients were applied to metagenomic sequencing. Libraries were constructed and sequenced on Illumina Nova6000 platform at Novagene Co, Beijing, China. Bactopia Analysis Pipeline version 1.7.1 (University of Trento) was used for genome assembly of isolated strains. Then StrainSifter^27^ was used to calculate the single-nucleotide variant of the metagenomic sequences between isolated strains and nasopharyngeal swab, saliva, and fecal samples. SAMtools version 1.7 (Wellcome Trust Genome Campus) and BamTools version 2.4.0 (University of South Florida) with a defined pepline (across positions >0.1 ×) were applied to generate quantifiable single-nucleotide variations.^28,29^

Libraries were constructed and sequenced on Illumina NovaSeq 6000 platform. Fragments were mapped to the human genome (hg19) using Hisat2 version 2.0.5 (Johns Hopkins University), and gene abundance was reported as read counts.^30^ Differentially expressed genes were analyzed using the DESeq2 package version 1.30.1 (Bioconductor). A gene list was obtained from MSigDB version 7.4 (University of California San Diego and Broad Institute), containing 2328 concepts and 18 893 protein-coding genes. The pathway enrichment list was then generated by gene set enrichment analyses with R packages clusterProfiler, enrichplot, and fgsea (R Foundation).^31^ Through knowledge-based annotation, we refined pathways to 4 categories: metastasis, proliferation, immune response, and signal pathways. Microenvironment cell populations–counter immune estimation was used to assess immune infiltration.^32^ Hematoxylin and eosin and immunohistochemistry assays were used to analyze CD8^+^ T-cell infiltration with an anti-CD8^+^ antibody (Abcam). A full view of each slide was scanned and analyzed using HALO image software version 3.3.2541.420 (lndica Labs) (eMethods in Supplement 1). According to the ImmPort database,^33^ differentially expressed genes with adjusted Benjamini-Hochberg *P* < .05 were annotated, and Spearman correlations between these genes and intratumoral bacteria were calculated.

### Statistical Analysis

The primary end point was disease-free survival, and the secondary end points included distant metastasis–survival and overall survival. Disease-free survival was defined as the time from the first day of therapy to tumor relapse at any site or death from any cause, whichever occurred first; distant metastasis– survival to distant metastasis or non–cancer-specific death; and overall survival to death from any cause. Patients who were lost to follow-up or still alive without relapse were censored at the data of last follow-up. The paired Wilcoxon signed rank test was used to compare 2 paired groups. X-tile software version 3.6.1 (Yale University) was applied to obtain the optimal cutoff for dividing patients into high or low bacterial load groups.^34^ χ^2^ test or Fisher exact test were used to compare categorical variables. Survival probability was estimated by Kaplan-Meier method and compared by log-rank test, and hazard ratios (HRs) were calculated by univariable Cox analysis. Multivariable Cox analysis with backward selection was used to identify independent factors, and sex, age, stage, pathological type, intratumoral bacterial load, and chemotherapy were used as covariates. All analyses were performed by SPSS version 22.0 (IBM) and R version 4.0.3 (R Foundation) with 2-tailed tests, and *P* < .05 was considered significant.

## Results

### Patient Characteristics

A total of 802 patients with NPC (mean [SD] age, 46.2 [10.6] years; 594 [74.1%] male) were enrolled in this study (Figure 1). In the discovery cohort, 96 patients with or without posttreatment tumor relapse were matched by sex, age, tumor node metastasis stage, and treatment modalities (eTable 1 in Supplement 1). Patient characteristics of training, internal validation, and external validation cohorts are shown in the Table. The median (IQR) follow-up was 83.1 (60.3-94.9) months in the training cohort, 99.0 (60.1-108.5) months in the internal validation cohort, and 89.7 (56.6-97.3) months in the external validation cohort.

**Table.** Clinical Characteristics of Patients in the Training, Internal Validation, and External Validation Cohorts

Characteristic	Training cohort (n = 241)			Internal validation cohort (n = 233)			External validation cohort (n = 232)		
	No. of patients	Low load (%)	High load (%)	No. of patients	Low load (%)	High load (%)	No. of patients	Low load (%)	High load (%)
Sex									
Female	54	45 (23)	9 (19)	66	52 (28)	14 (28)	67	43 (26)	24 (35)
Male	187	148 (77)	39 (81)	167	131 (72)	36 (72)	165	121 (74)	44 (65)
Age, y									
<45	125	107 (55)	18 (38)	112	85 (46)	27 (54)	87	64 (39)	23 (34)
≥45	116	86 (45)	30 (63)	121	98 (54)	23 (46)	145	100 (61)	45 (66)
T stage									
T1	25	22 (11)	3 (6)	24	20 (11)	4 (8)	35	22 (13)	13 (19)
T2	39	32 (17)	7 (15)	58	45 (25)	13 (26)	119	87 (53)	32 (47)
T3	128	105 (54)	23 (48)	86	65 (35)	21 (42)	46	32 (20)	14 (21)
T4	49	34 (18)	15 (31)	65	53 (29)	12 (24)	32	23 (14)	9 (13)
N stage									
N0	29	26 (13)	3 (6)	41	29 (16)	12 (24)	38	28 (17)	10 (15)
N1	107	84 (44)	23 (48)	116	98 (54)	18 (36)	71	51 (31)	20 (29)
N2	65	53 (27)	12 (25)	50	37 (20)	13 (26)	97	68 (42)	29 (43)
N3	40	30 (16)	10 (21)	26	19 (10)	7 (14)	26	17 (10)	9 (13)
TNM stage									
I	10	8 (4)	2 (4)	7	4 (2)	3 (6)	2	2 (1)	0
II	30	23 (12)	7 (15)	51	44 (24)	7 (14)	58	44 (27)	14 (21)
III	119	101 (52)	18 (37)	93	70 (38)	23 (46)	114	78 (48)	36 (53)
IV	82	61 (32)	21 (44)	82	65 (36)	17 (34)	58	40 (24)	18 (26)
WHO pathological type									
Undifferentiated nonkeratinizing	237	190 (98)	47 (98)	226	177 (97)	49 (98)	212	147 (90)	65 (96)
Differentiated nonkeratinizing	4	3 (2)	1 (2)	7	6 (3)	1 (2)	20	17 (10)	3 (4)
Plasma EBV-DNA									
<2000 Copies/mL	108	92 (48)	16 (33)	NA	NA	NA	NA	NA	NA
≥2000 Copies/mL	133	101 (52)	32 (67)	NA	NA	NA	NA	NA	NA
Chemotherapy									
Yes	223	180 (93)	43 (90)	174	137 (75)	37 (74)	202	145 (88)	57 (84)
No	18	13 (7)	5 (10)	59	46 (25)	13(26)	30	19 (12)	11 (16)

Abbreviations: EBV, Epstein-Barr virus; NA, not available; TNM, tumor node metastasis; WHO, World Health Organization.

### Microbiota Existed in NPC Tissues and Was Associated With Tumor Relapse

**Figure 2.**
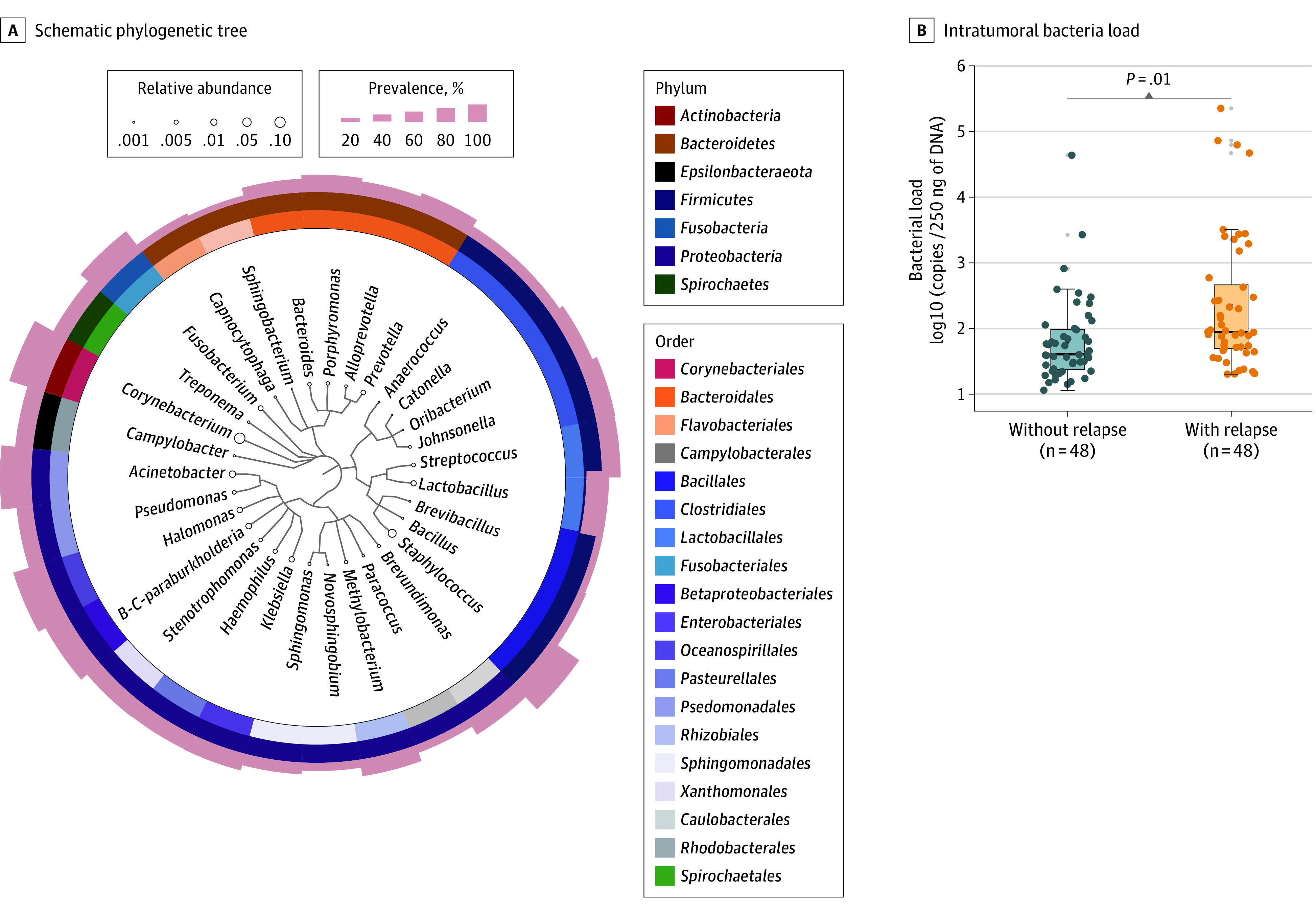
Microbiota in Nasopharyngeal Carcinoma (NPC) Tissues Associated With Tumor Relapse A, Schematic phylogenetic tree depicting the representative bacterial genera of 48 paired NPC tissues with or without tumor relapse based on 16S rRNA sequencing. The different colors and shades in the circles indicate the classifications of bacteria at the order (inner circle) and phylum (middle circle) levels. The size of the circle represents the relative abundance of genus levels, and the height of the shadow (outer circle) represents the level of prevalence within NPC tumors. B, The intratumoral bacterial load of 48 paired patients with NPC with or without tumor relapse was assessed by quantitative polymerase chain reaction. The comparison was performed with paired Wilcoxon signed rank test. *B-C* indicates *Burkholderia-Caballeronia*.

After filtering contaminants (eFigure 4 in Supplement 1), representative bacterial genera were screened and used to construct a schematic phylogenetic tree. The results showed that *Proteobacteria* accounted for the highest proportion among the 8 phyla, with *Corynebacterium* and *Staphylococcus* occupying the largest relative abundance (12.9% and 7.4%, respectively) and prevalence (81.0% and 76.0%, respectively) at the genus level (Figure 2A). Notably, patients with tumor relapse exhibited significantly enhanced α diversity (Shannon and InvSimpson index) compared with those without tumor relapse, while β diversity showed nondifferential clustering of amplicon sequence variants (eFigure 5 in Supplement 1). Moreover, tumors from patients with relapse exhibited a significant increase in *Prevotella* and *Porphyromonas* levels (eFigure 6 in Supplement 1). A significantly higher bacterial load was found in patients with tumor relapse (Figure 2B; eFigure 7 in Supplement 1). Furthermore, 16S rRNA gene probed fluorescence in situ hybridization assay verified that the bacteria presented within NPC tissues (eFigure 8 in Supplement 1), which could also be visualized by immunohistochemistry staining against bacterial lipopolysaccharide antigen (eFigure 9 in Supplement 1).

### Intratumoral Bacteria Mainly Originated From the Nasopharyngeal Microbiota

Bacteria from 15 of 20 NPC tissues were successfully cultivated and a total of 29 strains were identified (eFigure 10 in Supplement 1). Metagenomic sequencing was performed to analyze the single-nucleotide variations between these representative bacteria from NPC tissues and strains from suspected sites. By comparing strains among matched samples from the same patient, the 29 strains had single-nucleotide variant similarities with bacteria in either nasopharynx (69.0%), oral cavity (24.1%), or gut (6.9%), indicating that the intratumoral bacteria mainly originated from the nasopharyngeal microbiota (eFigure 11 in Supplement 1).

### High Intratumoral Bacterial Load Was Associated With Poor Prognosis in Patients With NPC

The intratumoral bacterial loads were then quantified in 706 patients from the 3 cohorts (eFigure 12 in Supplement 1). We first applied X-tile plots to obtain an optimal cutoff (206.4) for separating patients into low- or high-load groups in the training cohort (eFigure 13 in Supplement 1). This cutoff allotted 193 of 241 patients (80.1%) to the low-load group and 48 (19.9%) to the high-load group. In the internal validation cohort, 183 patients (78.5%) and 50 patients (21.5%) were respectively classified into the low- or high-load groups with the same cutoff developed in the training cohort. In the external validation cohort, there were 164 patients (70.7%) and 68 patients (29.3%) in the low- and high-load groups, respectively.

**Figure 3.**
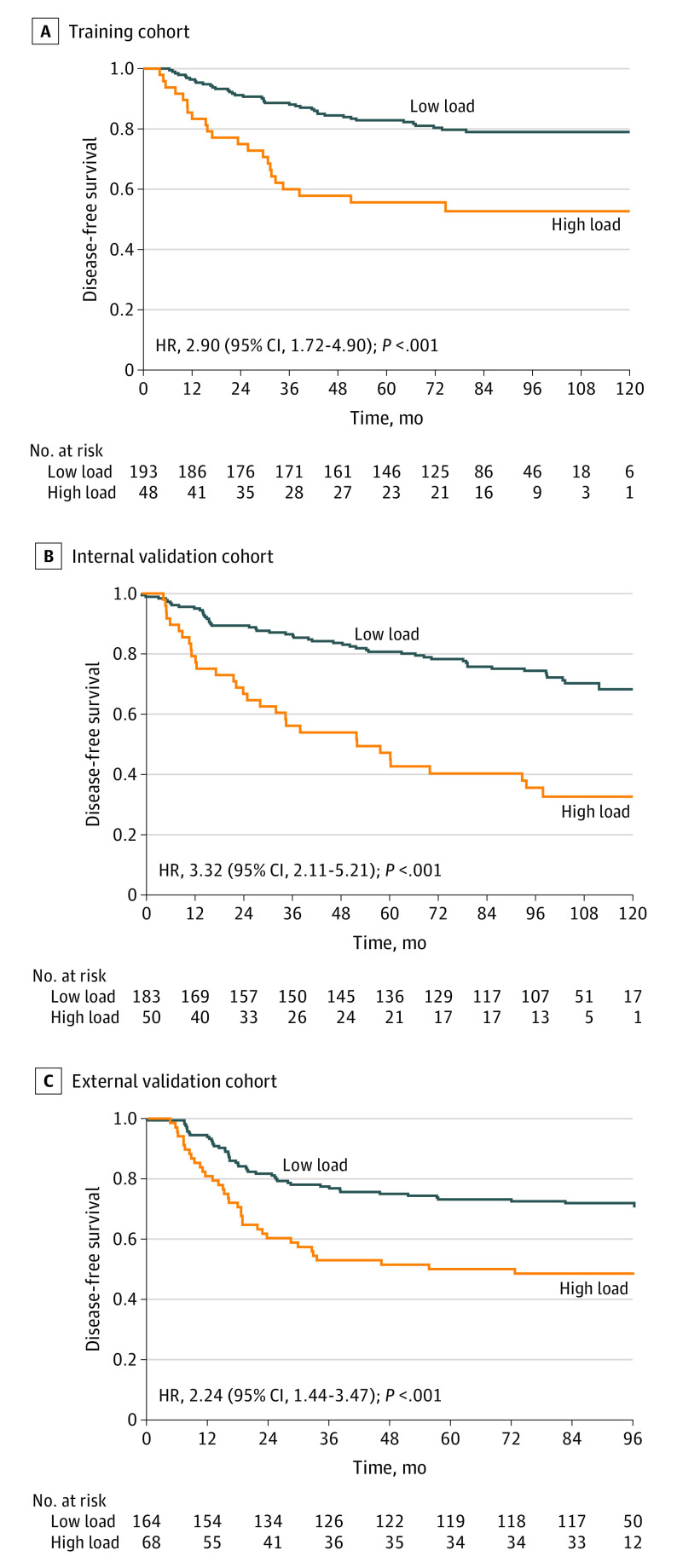
High Intratumoral Bacterial Load Associated With Poor Prognosis in Patients With Nasopharyngeal Carcinoma (NPC) A-C, Kaplan-Meier curves of disease-free survival for the training cohort (n = 241), the internal validation cohort (n = 233), and the external validation cohort (n = 232). We calculated *P* values using unadjusted log-rank test and hazard ratios (HRs) and 95% CIs using univariable Cox regression analysis.

Survival analysis demonstrated that patients with a high bacterial load had worse disease-free survival in the training cohort (HR, 2.90; 95% CI, 1.72-4.90; *P* < .001), the internal validation cohort (HR, 3.32; 95% CI, 2.11-5.21; *P* < .001), and the external validation cohort (HR, 2.24; 95% CI, 1.44-3.47; *P* < .001) (Figure 3). Similar results were found for distant metastasis–free survival and overall survival (eFigure 14 in Supplement 1).

Univariable regression analysis revealed that the intratumoral bacterial load was significantly associated with disease-free survival in all 3 cohorts (eFigure 15 in Supplement 1). After adjusting for other clinical characteristics, multivariable Cox regression analysis identified that the intratumoral bacterial load remained a strong independent prognostic indicator for disease-free survival in the training cohort (HR, 2.35; 95% CI, 1.37-4.01; *P* = .002), as well as in the internal validation cohort (HR, 3.54; 95% CI, 2.24-5.61; *P* < .001) and the external validation cohort (HR, 2.17; 95% CI, 1.40-3.37; *P* < .001). Similar results were obtained for distant metastasis–free survival and overall survival (eTables 2-4 in Supplement 1).

### Intratumoral Bacteria Load Was Negatively Associated With T-Lymphocyte Infiltration

**Figure 4.**
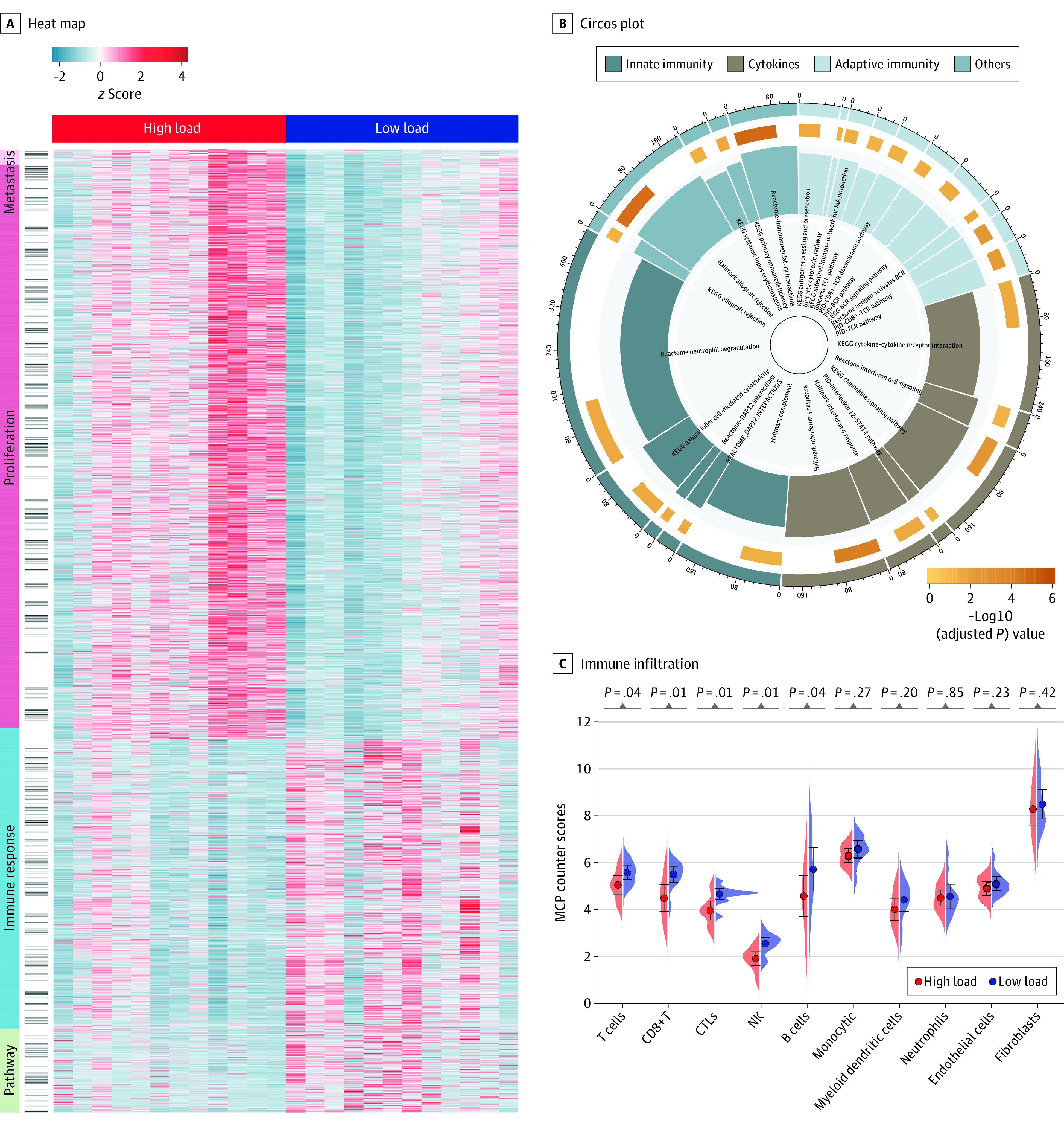
Intratumoral Bacteria Load Negatively Associated With T-Lymphocyte Infiltration A, Heatmap showing molecular features of patients with nasopharyngeal carcinoma with a high or low bacterial load through gene set enrichment analyses (GSEA), with knowledge-based annotation of 4 main types: metastasis, proliferation, immune response, and other pathways. B, Circos plot showing the significantly enriched immunological pathways in tumors with a low bacterial load. The outermost circle represents the number of genes contained in the labeled pathway. The size of the second outer circle represents the number of genes enriched by GSEA, and the color depth represents the adjusted Benjamini-Hochberg *P* value. The inner bar plot shows the normalized enrichment score. Different colors in the circle represent the category of pathways. C, The immune infiltration of patients with a high or low bacterial load estimated by the microenvironment cell populations (MCP)–counter algorithm. The comparison was performed with paired Wilcoxon signed rank test. BCR indicates B-cell receptor; CTLs, cytotoxic T-lymphocytes; KEGG, Kyoto Encyclopedia of Genes and Genomes; NK, natural killer; PID, Pathway Interaction Database; TCR, T-cell receptor.

Twelve paired NPC tissues with high or low bacteria load were subjected to host transcriptional analysis (eTable 5 in Supplement 1). As expected, typical proliferative gene sets of cell-cycle pathways (such as *MYC* and *PLK1*) and metastasis-associated pathways were identified to be enriched in tumors with a high bacterial load. Conversely, tumors with a low bacterial load were characterized by an active immune response, including T-cell receptor, B-cell receptor, and interferon signals, as well as enhanced levels of several signal pathways, such as O-linked glycosylation (Figure 4A and B; eTable 6 in Supplement 2).

Tumor immune infiltration analysis showed that tumors with low bacterial load were infiltrated with more CD8^+^ T, natural killer, and cytotoxic T-lymphocyte than those with high bacterial load. Digital pathology analysis confirmed that tumors with low bacterial load were significantly associated with an increase in CD8^+^ T cells (Figure 4C; eFigure 16 in Supplement 1). We further annotated the differential immunological gene sets and analyzed their correlation with intratumoral bacteria in NPC. The results revealed that the expression levels of most immune-associated genes, such as *CXCL13*, were negatively associated with the abundance of intratumoral bacteria such as *Porphyromonas* (eFigure 17 in Supplement 1).

## Discussion

In this multicenter retrospective cohort study, we present what is to our knowledge the first report to uncover the microbial landscape and its clinical implications in NPC. We found that microbiota presented within NPC tissues, and nasopharynx was the main origin of NPC intratumoral bacteria. Focusing on its critical involvement in NPC malignant behaviors, we noted that the intratumoral bacterial load served as an efficacious prognostic indicator.

Intratumoral bacteria has been forcefully explored within seven human cancer types according to a strict contamination control strategy, which was found to be associated with clinical features and had implications in prognosis.^16^ Tumors originating in the nasopharynx are unique, given the role of the nasopharynx as gatekeeper of the respiratory tract, and the potential impact of bacterial colonization on cancer development might be greatly underestimated. Here, we performed 16S rRNA sequencing to uncover the existence of microbiota. To address concerns regarding DNA contamination, a rigorous contamination removal procedure was implemented, and contaminants like *Mycoplasma* were successfully filtered out. We found that *Corynebacterium* and *Staphylococcus* were dominant in the microbial composition of NPC tissues, which was consistent with further strain isolation experiments. In contrast to high α diversity that is known to facilitate prognosis in pancreatic cancer,^17^ we observed a higher α diversity associated with NPC tumor relapse, which might be because of the caustic bacterial environment of NPC. The rarefaction curve showed that amplicon sequence variants sequenced from different individuals had strong heterogeneity, which was the main reason for the indistinctive separation trend within groups characterized by β diversity, suggesting that the absolute bacterial load might be a more decisive factor.

As the first study focusing on intratumoral bacteria of NPC, we uncovered that the bacterial load served as an independent prognostic factor. Bacterial load assessment based on quantitative polymerase chain reaction provides a promising and convenient resolution to investigate the significance of microbiota in NPC prognosis. A recent study^35^ reports that tumor-resident intracellular microbiota promoted breast cancer metastatic colonization, providing biological foundation for the application of intratumoral bacteria as prognostic indicator. Of note, current research supports that the disruption of microorganisms affects the response of antitumor therapy considerably.^36^ In addition, it has been reported that butyrate produced by *Porphyromonas gingivalis* may take part in the regulation of histone acetylation and the reactivation of Epstein-Barr virus,^37^ supporting the potential crosstalk between bacteria and Epstein-Barr virus. We anticipate that further exploration of intratumoral bacteria and Epstein-Barr virus will furnish NPC with a new regulatory mechanism with respect to its sophisticated biological process, which will pave a path to the era of microbiome-driven precision medicine and ultimately ameliorate outcomes in patients with NPC.

Owing to technical limitations, attempts at metagenomic sequencing have failed because this process captures more than 99% of host information, which makes the source of intratumoral bacteria a pending issue. Analysis of representative strains is a good alternative, as demonstrated in tracing the origin of blood pathogens.^27^ Here, we isolated 29 representative strains, most of which were *Staphylococcus epidermidis* and *Staphylococcus aureus*. This was consistent with the 16S rRNA sequencing data that showed the highest abundance of *Staphylococcus* genus, indicating the reliability of traceability analysis. Based on this, we observed that the NPC intratumoral bacteria mainly originated from the nasopharynx, and a small portion came from the oral cavity and intestine, suggesting multiple sources of intratumoral bacteria and a complex regulatory association among microbiota across different physiological sites in NPC.

Characterizing the tumor microbiome and its underlying mechanism is of great interest, as the incidences of multiple cancers are now attributed to infectious agents.^38^ Owing to their proinflammatory properties, *Helicobacter pylori* and *Fusobacterium nucleatum* are well-known motivators to gastric and colorectal cancers.^39,40^ Enhanced level of interleukin-17 secreted by macrophages can strengthen the activity of myeloid-derived suppressor cells, leading to a dysfunction of T and natural killer cells.^41^ Consistent with this, our study revealed a decreased CD8^+^ T infiltration in NPC tumors with high bacterial load. In addition to immunosuppressive feature, we observed that patients with high bacterial load exhibited significant cell cycle dependent proliferation characteristics, which may be mediated by mitogen-activated protein kinase cascades induced by bacteria via the toll-like and nucleotide-binding oligomerization domain–like receptors.^42^ Together, these potential mechanisms suggest that NPC microbiome might reinforce disease progression by modulating pathways both within tumor and immune microenvironment.

### Limitations

There are some limitations in this study. First, owing to technical limitations, especially in tumors with relatively low biomass, it was impossible to achieve complete microbial genome by metagenomic sequencing. In addition, although we observed a negative association between high bacterial load and T-lymphocyte infiltration, the relationship and underlying mechanisms behind this association need to be further explored. Furthermore, the interaction between intratumoral bacteria and Epstein-Barr virus warrants in-depth exploration.

## Conclusions

To our knowledge, this cohort study is the first with a large sample size to evaluate the microbial profiles in NPC tumors with different prognoses. The findings emphasize intratumoral bacterial load as a promising prognostic indicator in NPC.

## Notes

Supplement 1. eMethods eTable 1. Clinical characteristics of 48 paired NPC patients with or without tumour relapse eTable 2. Multivariable analysis of prognostic factors in the training cohort eTable 3. Multivariable analysis of prognostic factors in the internal validation cohort eTable 4. Multivariable analysis of prognostic factors in the external validation cohort eTable 5. Clinical characteristics of 12 paired NPC patients with high or low intratumoural bacterial load eFigure 1. Contamination removal procedure (CR procedure) for 16S rRNA sequencing eFigure 2. The rarefaction curve generated based on 16S rRNA sequencing data after the CR procedure eFigure 3. Establishment of a standard curve for quantitative PCR eFigure 4. Depletion of ASV counts and the top 20 representative genera pre and post–CR procedure in control and tissue samples eFigure 5. Diversity measurements between 48 paired NPC patients with or without tumour relapse eFigure 6. Differences in the predominance of bacterial communities (with the top 15 taxa in relative abundance) were characterized at the phylum, class, order, family and genus levels between 48 paired NPC patients with or without tumour relapse eFigure 7. DNA extraction controls were included to assess the bacterial loads in 48 paired NPC patients eFigure 8. Establishment of 16S rRNA gene-based FISH experiments eFigure 9. Immunohistochemical staining against lipopolysaccharide antigen in NPC tissues eFigure 10. Isolation of bacterial colonies from NPC tissues eFigure 11. Intratumoural bacteria in NPC mainly originate from the nasopharynx eFigure 12. DNA extraction and paraffin controls were included to assess bacterial loads in three cohorts eFigure 13. Determination of the cutoff point (206.4) for bacterial load using X-tile in the training cohort eFigure 14. High intratumoural bacterial load predicts poor prognosis of NPC patients eFigure 15. Univariable Cox analysis of intratumoural bacterial load and clinical characteristics with disease-free survival eFigure 16. CD8+ T cell infiltration was validated in 12 pairs of patients with high or low bacterial load eFigure 17. Network analysis based on the Spearman correlation coefficient of the microbiome and the differentially immunologically expressed genes

Supplement 2. eTable 6
