# Supplementary material for: Association of Intratumoral Microbiota With Prognosis in Patients With Nasopharyngeal Carcinoma From 2 Hospitals in China
Source: JAMA Oncol. 2022 Jul 14;8(9):1301–9. doi: 10.1001/jamaoncol.2022.2810 (PMC9284409; doi:10.1001/jamaoncol.2022.2810)
Supplement: Supplement 1. — eMethods eTable 1. Clinical characteristics of 48 paired NPC patients with or without tumour relapse eTable 2. Multivariable analysis of prognostic factors in the training cohort eTable 3. Multivariable analysis of prognostic factors in the internal validation cohort eTable 4. Multivariable analysis of prognostic factors in the external validation cohort eTable 5. Clinical characteristics of 12 paired NPC patients with high or low intratumoural bacterial load eFigure 1. Contamination removal procedure (CR procedure) for 16S rRNA sequencing eFigure 2. The rarefaction curve generated based on 16S rRNA sequencing data after the CR procedure eFigure 3. Establishment of a standard curve for quantitative PCR eFigure 4. Depletion of ASV counts and the top 20 representative genera pre and post–CR procedure in control and tissue samples eFigure 5. Diversity measurements between 48 paired NPC patients with or without tumour relapse eFigure 6. Differences in the predominance of bacterial communities (with the top 15 taxa in relative abundance) were characterized at the phylum, class, order, family and genus levels between 48 paired NPC patients with or without tumour relapse eFigure 7. DNA extraction controls were included to assess the bacterial loads in 48 paired NPC patients eFigure 8. Establishment of 16S rRNA gene-based FISH experiments eFigure 9. Immunohistochemical staining against lipopolysaccharide antigen in NPC tissues eFigure 10. Isolation of bacterial colonies from NPC tissues eFigure 11. Intratumoural bacteria in NPC mainly originate from the nasopharynx eFigure 12. DNA extraction and paraffin controls were included to assess bacterial loads in three cohorts eFigure 13. Determination of the cutoff point (206.4) for bacterial load using X-tile in the training cohort eFigure 14. High intratumoural bacterial load predicts poor prognosis of NPC patients eFigure 15. Univariable Cox analysis of intratumoural bacterial load and clinical characteristics with disease-free [file jamaoncol-e222810-s001.pdf]

Supplemental Online Content

Qiao H, Tan X-R, Li H, et al. Association of intratumoral microbiota with prognosis in patients with nasopharyngeal carcinoma from 2 hospitals in China. *JAMA Oncol*. Published online July 14, 2022. doi:10.1001/jamaoncol.2022.2810

eMethods

eTable 1. Clinical characteristics of 48 paired NPC patients with or without tumor relapse

eTable 2. Multivariable analysis of prognostic factors in the training cohort

eTable 3. Multivariable analysis of prognostic factors in the internal validation cohort

eTable 4. Multivariable analysis of prognostic factors in the external validation cohort

eTable 5. Clinical characteristics of 12 paired NPC patients with high or low intratumoral bacterial load

eFigure 1. Contamination removal procedure for 16S rRNA sequencing

eFigure 2. The rarefaction curve generated based on 16S rRNA sequencing data after the contamination removal procedure

eFigure 3. Establishment of a standard curve for quantitative PCR

eFigure 4. Depletion of ASV counts and the top 20 representative genera pre and post–contamination removal procedure in control and tissue samples

eFigure 5. Diversity measurements between 48 paired NPC patients with or without tumor relapse

eFigure 6. Differences in the predominance of bacterial communities (with the top 15 taxa in relative abundance) were characterized at the phylum, class, order, family and genus levels between 48 paired NPC patients with or without tumor relapse

eFigure 7. DNA extraction controls were included to assess the bacterial loads in 48 paired NPC patients

eFigure 8. Establishment of 16S rRNA gene-based fluorescence in situ hybridization experiments

eFigure 9. Immunohistochemical staining against lipopolysaccharide antigen in NPC tissues

eFigure 10. Isolation of bacterial colonies from NPC tissues

eFigure 11. Intratumoral bacteria in NPC mainly originate from the nasopharynx

© 2022 Qiao H et al. *JAMA Oncology.*

eFigure 12. DNA extraction and paraffin controls were included to assess bacterial loads in three cohorts

eFigure 13. Determination of the cutoff point (206.4) for bacterial load using X-tile in the training cohort

eFigure 14. High intratumoral bacterial load predicts poor prognosis of NPC patients

eFigure 15. Univariable Cox analysis of intratumoral bacterial load and clinical characteristics with disease-free survival

eFigure 16. CD8+ T cell infiltration was validated in 12 pairs of patients with high or low bacterial load

eFigure 17. Network analysis based on the Spearman correlation coefficient of the microbiome and the differentially immunologically expressed genes

This supplemental material has been provided by the authors to give readers additional information about their work.

© 2022 Qiao H et al. *JAMA Oncology.*

eMethods

Contamination removal procedure

The previous detailed framework for dealing with contamination in low biomass samples named the RIDE checklist was used to streamline dealing with the field's contamination.[1] The process of the contamination-removal procedure (CR procedure) is described in eFigure 1. Specifically, amplicon sequence variants (ASVs) containing either mitochondria or chloroplasts originating from the host in taxonomic annotation were removed by KneadData (filter 1).[2] To prevent a false-positive rate, we set up three different processing batch types: sequencing library batch (filter 2), DNA extraction batch (filter 3), and PCR amplification batch (filter 4). The resulting bacterial signature list was filtered to remove the singleton ASV (filter 5) and further filtered to retain the ASVs present in at least two patients (filter 6). We employed the nonparametric, exact, binomial test (setting .01 as the probability for null hypothesis) on each taxon's prevalence, x=number of successes in the sample and n=sample size to the background and *P*= taxon prevalence in the relevant batch controls. Only taxa that passed a *P* value cutoff of .05 in all batch comparisons were considered in the next analysis stage.

Digital pathology analysis

The digital pathology analysis was conducted using HALO image software version 3.3.2541.420 (lndica Labs) with the following five steps: (1) Registration of sequential tumor sections and alignment of tissue samples using the "Landmarks" function; (2) Two pathologists annotated the tumor areas manually on each hematoxylin and eosin slide and synchronised it on immunohistochemistry slides; (3) Nuclei segmentation by a function implemented in the multiplex immunohistochemistry features; (4) Automated classification of cells with positive staining in total areas and tumor areas; (5) Positive cells in total and tumor areas were counted and characterized by density of positive cells per mm^2.

References:

1. Eisenhofer R, Minich JJ, Marotz C, Cooper A, Knight R, Weyrich LS. Contamination in Low Microbial Biomass Microbiome Studies: Issues and Recommendations. *Trends Microbiol*. 2019;27(2):105-117. doi:10.1016/j.tim.2018.11.003
2. Pereira-Marques J, Hout A, Ferreira RM, et al. Impact of host DNA and sequencing depth on the taxonomic resolution of whole metagenome sequencing for microbiome analysis. *Front Microbiol*. 2019;10:1277. doi:10.3389/fmicb.2019.01277

eTable 1. Clinical characteristics of 48 paired NPC patients with or without tumor relapse

	Patients without tumor relapse	Patients with tumor relapse	*P* value
**Sex**			
Female	11 (23)	10 (21)	.81
Male	37 (77)	38 (79)	
**Age/years**			
<45	20 (42)	17 (35)	.53
≥45	28 (58)	31 (65)	
**T stage**			
T1	1 (2)	1(2)	.48
T2	6 (13)	2 (4)	
T3	26 (54)	26 (54)	
T4	15 (31)	19 (40)	
**N stage**			
N0	0 (0)	1 (2)	.39
N1	14 (29)	11 (22)	
N2	16 (33)	18 (38)	
N3	18 (38)	18 (38)	
**TNM stage**			
Ⅲ	19 (40)	15 (31)	.52
Ⅳ	29 (60)	33 (69)	
**Chemotherapy**			
yes	48 (100)	47 (98)	1.00
no	0 (0)	1 (2)	

TNM indicates tumor node metastasis.

eTable 2. Multivariable analysis of prognostic factors in the training cohort[a]

	Multivariate analysis		
**Variable**	**HR**	**95%CI**	***P* value**
**Disease-free survival**			
Age (≥45 vs. <45)	2.07	1.23-3.50	.007
EBV-DNA (≥ 2000 vs. <2000)	2.02	1.09-3.74	.03
Intratumoral bacterial load (high vs. low)	2.35	1.37-4.01	.002
**Distant metastasis-free survival**			
Age (≥45 vs. <45)	2.73	1.29-5.76	.009
EBV-DNA (≥ 2000 vs. <2000)	2.76	1.24-6.16	.01
Intratumoral bacterial load (high vs. low)	2.55	1.26-5.17	.009
**Overall survival**			
Age (≥45 vs. <45)	2.64	1.42-4.91	.002
EBV-DNA (≥ 2000 vs. <2000)	2.25	1.12-4.54	.02
Intratumoral bacterial load (high vs. low)	2.82	1.55-5.10	.001

HR indicates hazard ratio; EBV: Epstein-Barr virus.

[a] We calculated HRs and *p* values using an adjusted multivariable Cox proportional hazards regression model, including sex (male vs. female), age (≥ 45 years vs. < 45 years), TNM stage (III–IV vs. I–II), WHO pathological type (undifferentiated vs. differentiated), pretreatment plasma EBV-DNA (≥ 2000 copies/ml vs. < 2000 copies/ml), intratumoral bacterial load (high vs. low), and chemotherapy (no vs. yes) as covariates. We selected variables with the backwards stepwise approach. Only variables that were significantly associated with survival are presented.

eTable 3. Multivariable analysis of prognostic factors in the internal validation cohort[a]

	Multivariate analysis		
**Variable**	**HR**	**95%CI**	***P* value**
**Disease-free survival**			
Sex (male vs. female)	2.31	1.29-4.14	.005
TNM stage (III-IV vs. I-II)	2.24	1.21-4.16	.01
WHO type (undifferentiated vs. differentiated)	2.94	1.05-8.23	.04
Intratumoral bacterial load (high vs. low)	3.54	2.24-5.61	<.001
**Distant metastasis-free survival**			
Sex (male vs. female)	2.24	1.09-4.61	.03
Age (≥45 vs. <45)	1.96	1.11-3.46	.02
TNM stage (III-IV vs. I-II)	5.43	1.95-15.11	.001
WHO type (undifferentiated vs. differentiated)	3.21	1.12-9.17	.03
Intratumoral bacterial load (high vs. low)	4.40	2.57-7.55	<.001
**Overall survival**			
Sex (male vs. female)	2.52	1.29-4.94	.007
Age (≥45 vs. <45)	1.66	1.02-2.69	.04
TNM stage (III-IV vs. I-II)	3.98	1.82-8.73	.001
Intratumoral bacterial load (high vs. low)	3.89	2.41-6.27	<.001

HR indicates hazard ratio; TNM, tumor node metastasis; WHO, World Health Organization.

[a] We calculated HRs and *p* values using an adjusted multivariable Cox proportional hazards regression model, including sex (male vs. female), age (≥ 45 years vs. < 45 years), TNM stage (III–IV vs. I–II), WHO pathological type (undifferentiated vs. differentiated), intratumoral bacterial load (high vs. low), and chemotherapy (no vs. yes) as covariates. We selected variables with the backwards stepwise approach. Only variables that were significantly associated with survival are presented.

eTable 4. Multivariable analysis of prognostic factors in the external validation cohort[a]

	Multivariate analysis		
**Variable**	**HR**	**95%CI**	***P* value**
**Disease-free survival**			
TNM stage (III-IV vs. I-II)	2.61	1.38-4.93	.003
Intratumoral bacterial load (high vs. low)	2.17	1.40-3.37	.001
**Distant metastasis-free survival**			
Intratumoral bacterial load (high vs. low)	2.54	1.40-4.60	.002
**Overall survival**			
TNM stage (III-IV vs. I-II)	2.91	1.45-5.84	.003
Intratumoral bacterial load (high vs. low)	2.12	1.33-3.37	.002

HR indicates hazard ratio; TNM, tumor node metastasis.

[a] We calculated HRs and *p* values using an adjusted multivariable Cox proportional hazards regression model, including sex (male vs. female), age (≥ 45 years vs. < 45 years), TNM stage (III–IV vs. I–II), WHO pathological type (undifferentiated vs. differentiated), intratumoral bacterial load (high vs. low), and chemotherapy (no vs. yes) as covariates. We selected variables with the backwards stepwise approach. Only variables that were significantly associated with survival are presented.

eTable 5. Clinical characteristics of 12 paired NPC patients with high or low intratumoral bacterial load

	Patients with low bacterial load (%)	Patients with high bacterial load (%)	*P* value
**Sex**			
Female	2 (17)	4 (33)	.64
Male	10 (83)	8 (67)	
**Age/years**			
<45	6 (50)	6 (50)	1.00
≥45	6 (50)	6 (50)	
**T stage**			
T2	4 (33)	1 (8)	.25
T3	2 (17)	5 (42)	
T4	6 (50)	6 (50)	
**N stage**			
N1	3(25)	3 (25)	.35
N2	3 (25)	6 (50)	
N3	6 (50)	3 (25)	
**TNM stage**			
Ⅲ	1 (8)	5 (42)	.16
Ⅳ	11 (92)	7 (58)	
**Chemotherapy**			
yes	12 (100)	12 (100)	—

TNM indicates tumor-node-metastasis.

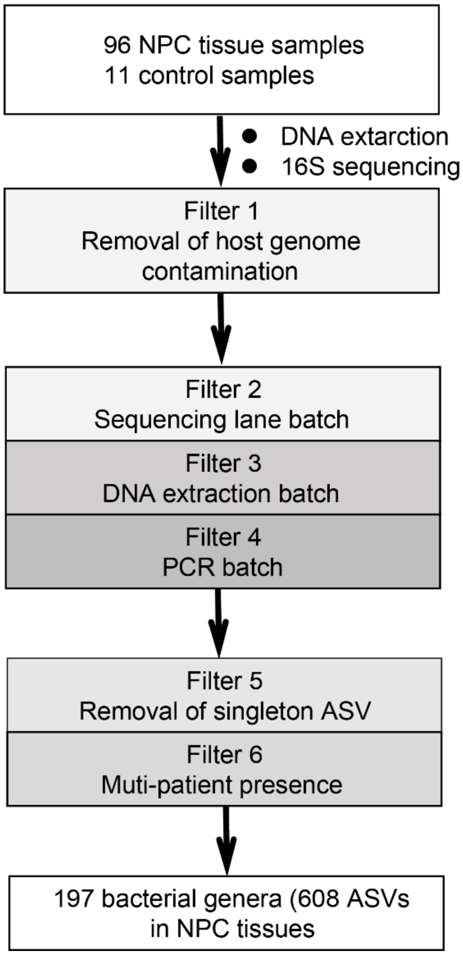

eFigure 1. Contamination removal procedure for 16S rRNA sequencing
PCR indicates polymerase chain reaction; ASV, amplicon sequence variants.

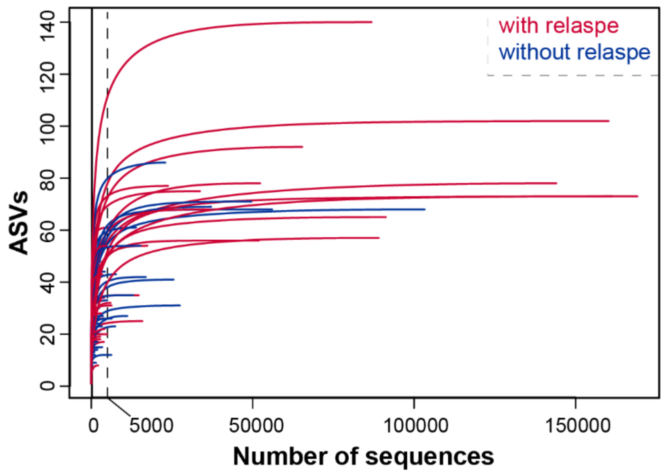

eFigure 2. The rarefaction curve generated based on 16S rRNA sequencing data after the contamination removal procedure

An even sampling depth of 5,000 sequences per sample was used for α and β diversities measurement.

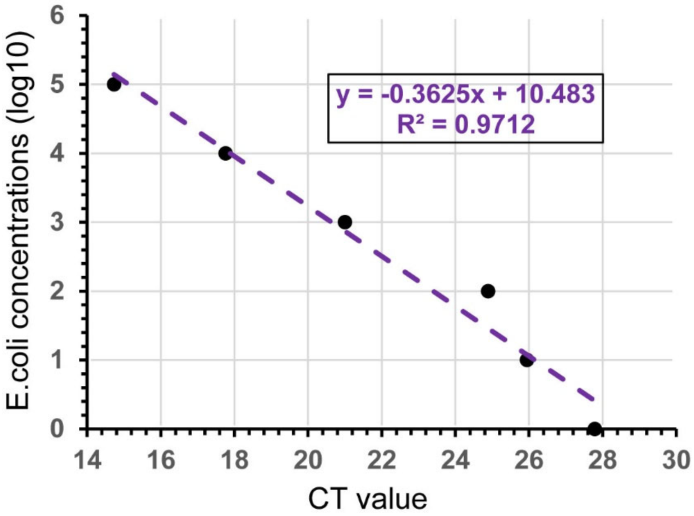

eFigure 3. Establishment of a standard curve for quantitative PCR

The x axis represents five 10-fold proportional dilutions of *E. coli* genomic DNA templates with an initial concentration of 6×10^5 copies/μl. The y axis represents CT values.

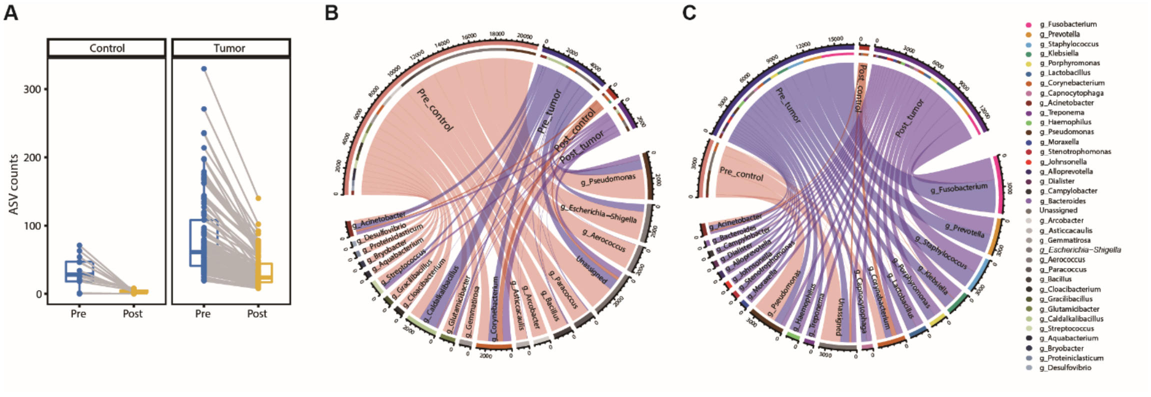

eFigure 4. Depletion of ASV counts and the top 20 representative genera pre and post–contamination removal procedure in control and tissue samples

(A) The contamination removal (CR)–procedure effectively filtered out the contaminant amplicon sequence variants (ASVs) in the control and tissue samples, and the grey line connect the same sample's observed ASVs counts pre (before) and post (after)–CR procedure. (B) The circos plot showed the changes of reads in control and tissue samples pre and post–CR procedure at the genus level (the top 20 genera in control samples pre–CR procedure). (C) Maintained ASVs in control and tissue samples pre and post–CR procedure at the genus level (the top 20 genera in tissue samples post–CR procedure). The spread values of the circos plot indicate the average number of reads in the group or genera, and the inner links show paired relations between the group and genera. "pre_control" and "post_control", "pre_tumor" and "post_tumor" represent control samples and tissue samples pre and post–CR procedure, respectively.

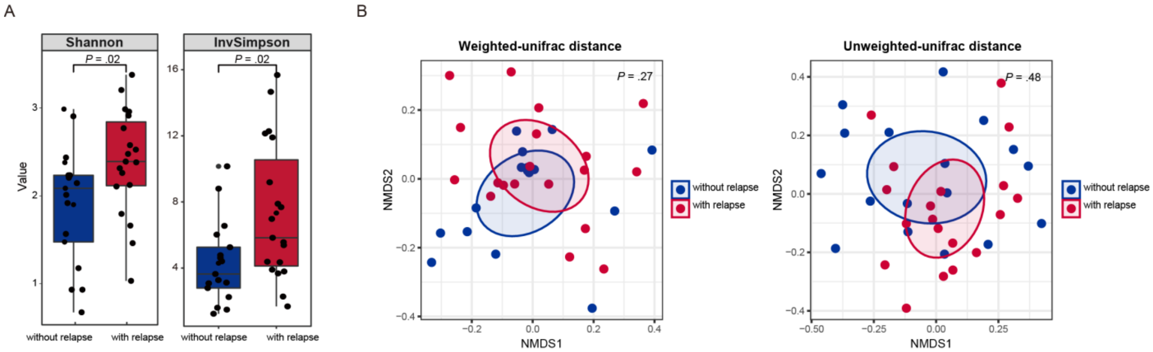

eFigure 5. Diversity measurements between 48 paired NPC patients with or without tumor relapse

(A) Alpha diversity (Shannon and InvSimpson index) revealed differences in community richness and evenness of bacteria between 48 paired NPC patients with or without tumor relapse. (B) Principal coordinate analysis (PCoA) using weighted-unifrac distance and unweighted-unifrac distance of beta diversity revealed diversity distance of bacteria between 48 paired NPC patients with or without tumor relapse.

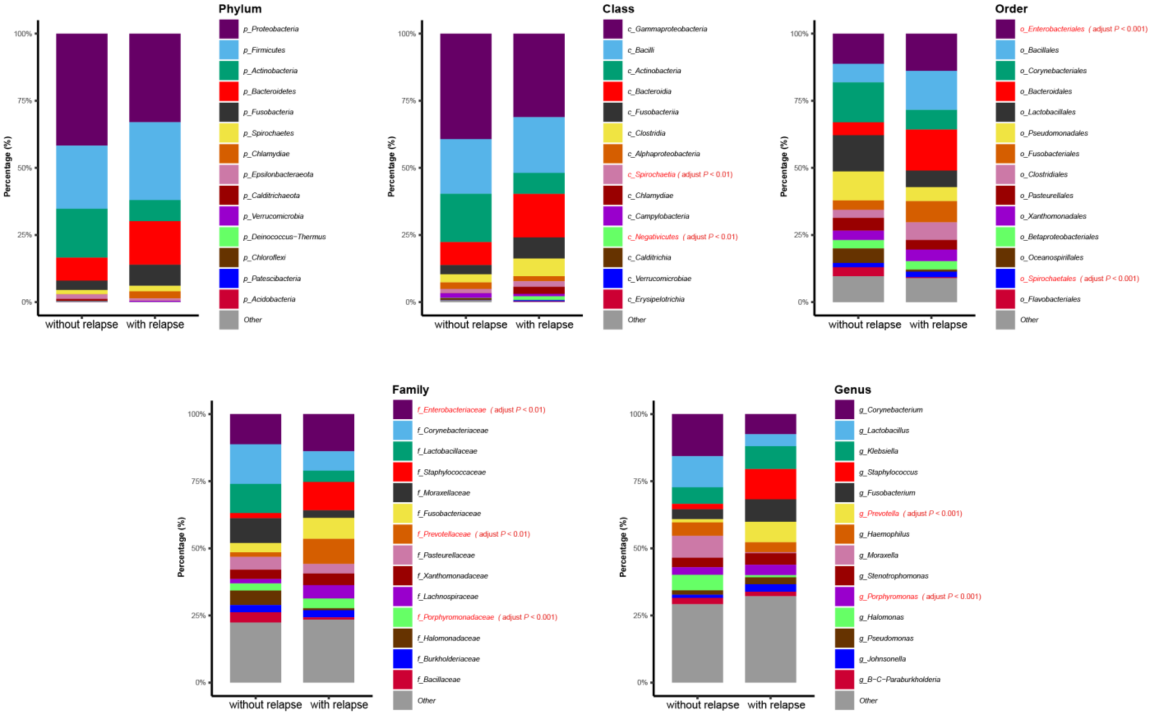

eFigure 6. Differences in the predominance of bacterial communities (with the top 15 taxa in relative abundance) were characterized at the phylum, class, order, family and genus levels between 48 paired NPC patients with or without tumor relapse

Differential taxa with adjusted Benjamini-Hochberg *P* < .05 are labelled in red.

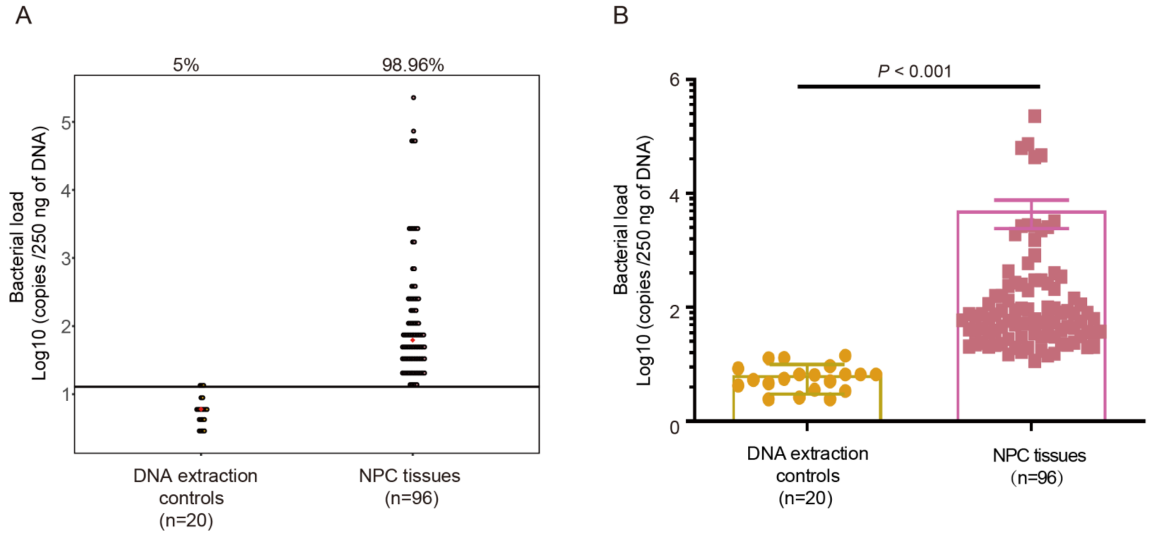

eFigure 7. DNA extraction controls were included to assess the bacterial loads in 48 paired NPC patients

(A) With 13 copies (CT value=28) as the detection limit, the positive detection rate in 48 paired NPC patients and DNA extraction controls were depicted. Black bar represents the detection limit. Red dots represent the median. (B) Bacterial load of the two groups was compared by paired Wilcoxon signed rank test.

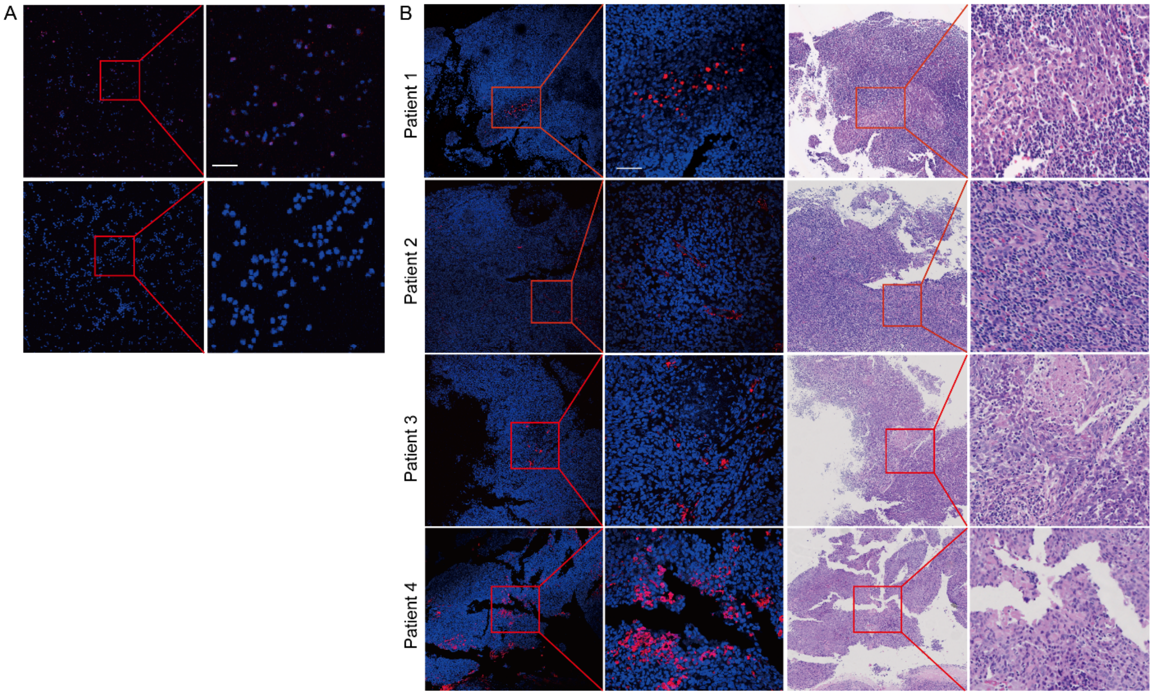

eFigure 8. Establishment of 16S rRNA gene-based fluorescence in situ hybridization experiments

(A) The NPC cell line HONE1 treated with *E. coli* (upper row) and untreated cells (lower row) were subjected to fluorescence in situ hybridization experiments as positive and negative control samples, respectively. High-magnification images of the boxed area are shown on the right (40× and 400×). (B) Representative staining of 16S rRNA probes in tissues of different NPC patients. The same NPC tissues were taken for sectioning, and the two adjacent tissues were stained with hematoxylin and eosin or fluorescence in situ hybridization probes. High-magnification images of the boxed area are shown on the right (40× and 400×). Scale bars, 50 μm.

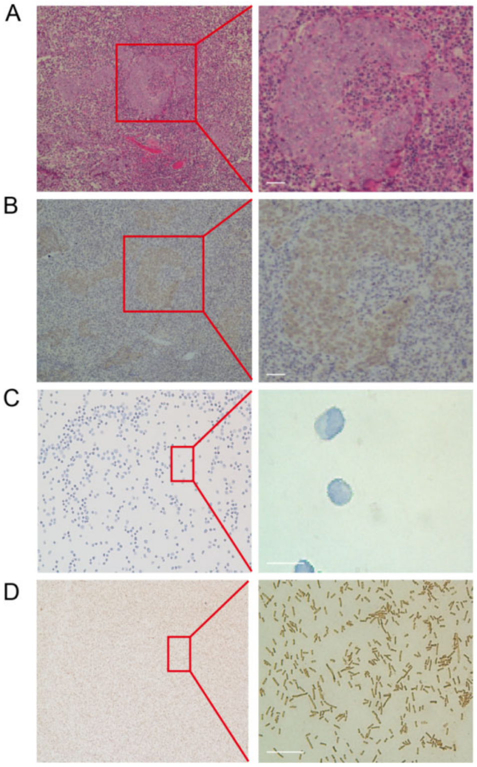

eFigure 9. Immunohistochemical staining against lipopolysaccharide antigen in NPC tissues

(A-B) Consecutive slices from NPC tissues were stained with lipopolysaccharide or H&E. High-magnification images of the boxed area are shown on the right (100× and 400×). (C-D) HONE1 cells and *E. coli* were stained with lipopolysaccharide and served as a negative or positive control. High-magnification images of the boxed area are shown on the right (40× and 400×). Scale bars, 20 μm.

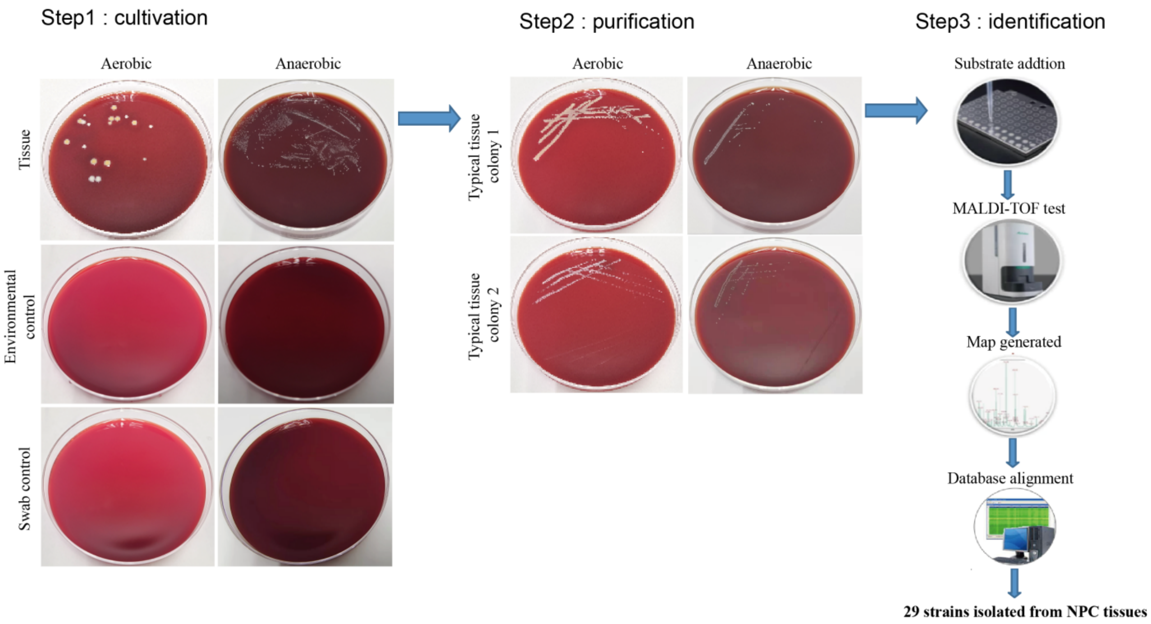

eFigure 10. Isolation of bacterial colonies from NPC tissues

Sterile forceps and scissors were used to leak the medial NPC tissues, and then intratumoral bacteria were dipped with sterile swabs, coated on blood agar plates, and cultured at 37 °C for 48 hour in aerobic (left) or anaerobic (right) conditions. Colonies were successfully obtained from 15 of 20 NPC tissues. Environmental and swab controls were set, and no colony growth was observed. Typical colonies were selected and further isolated by the tetrachzonal line method. For example, *Staphylococcus aureus* and *Staphylococcus epidermidis* were isolated under aerobic conditions, and *Cutibacterium acnes* and *Aerococcus viridans were* isolated under anaerobic conditions. A total of 29 strains were isolated and identified by MALDI-TOF mass spectrometry.

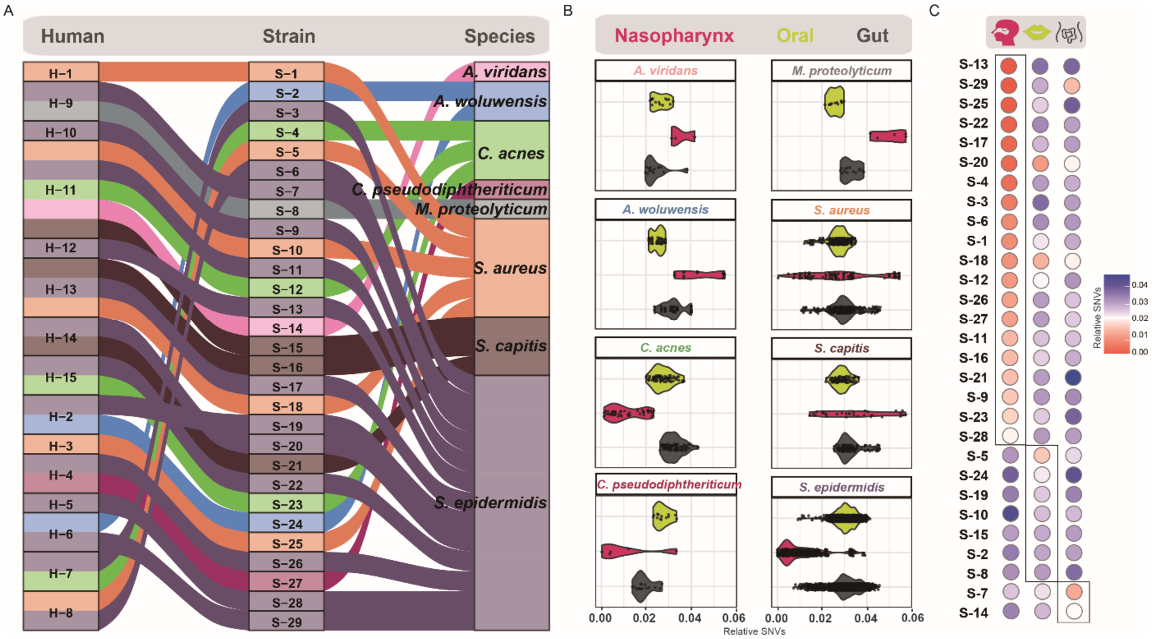

eFigure 11. Intratumoral bacteria in NPC mainly originate from the nasopharynx

(A) Sankey diagram summarizing the information of 29 isolated strains (middle column) from the corresponding 15 NPC tissues (left column) and eight species-level notes (right column). (B) Box plots depicting the similarities between the eight types of species isolated from NPC tissues and nasopharyngeal swab, saliva, and faecal samples. (C) Statistical results showing the relative single-nucleotide variants between isolated strains from NPC tissues and the matched nasopharyngeal swab, saliva, and faecal samples from the same patient. "Relative single-nucleotide variants" represents the relative coefficient of single-nucleotide variants.

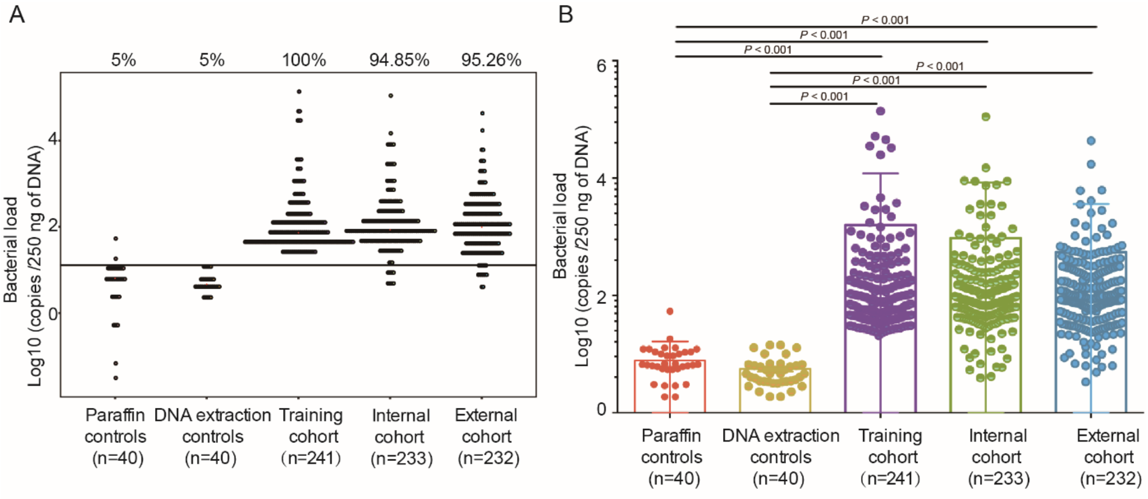

eFigure 12. DNA extraction and paraffin controls were included to assess bacterial loads in three cohorts

(A) With 13 copies (CT value=28) as the detection limit, the positive detection rate in three cohorts, DNA extraction and paraffin controls were depicted. Black bar represents the detection limit. Red dots represent the median. (B) Bacterial load of the five groups was compared by paired Wilcoxon signed rank test.

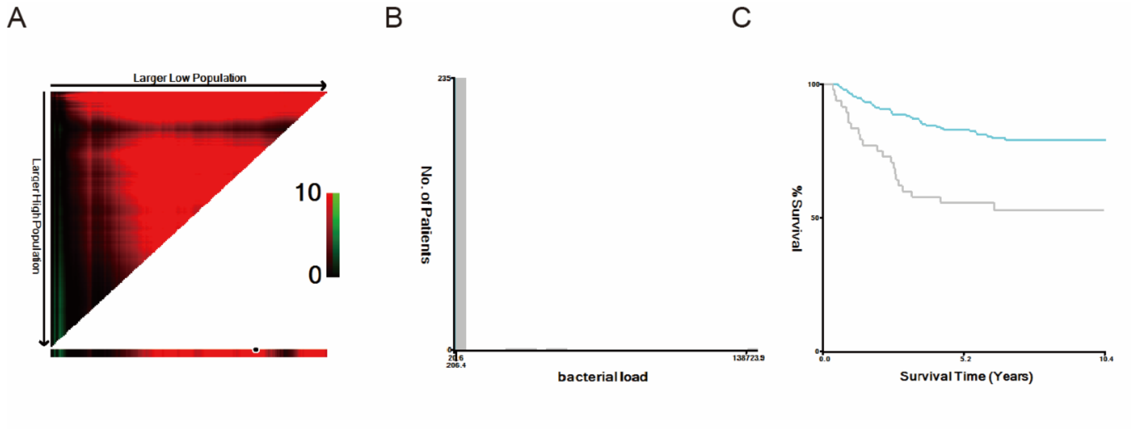

eFigure 13. Determination of the cutoff point (206.4) for bacterial load using X-tile in the training cohort

The colours in the plot represent the strength of the association at each division. Red indicates an inverse relationship between the risk score and disease-free survival, and green indicates a positive relationship.

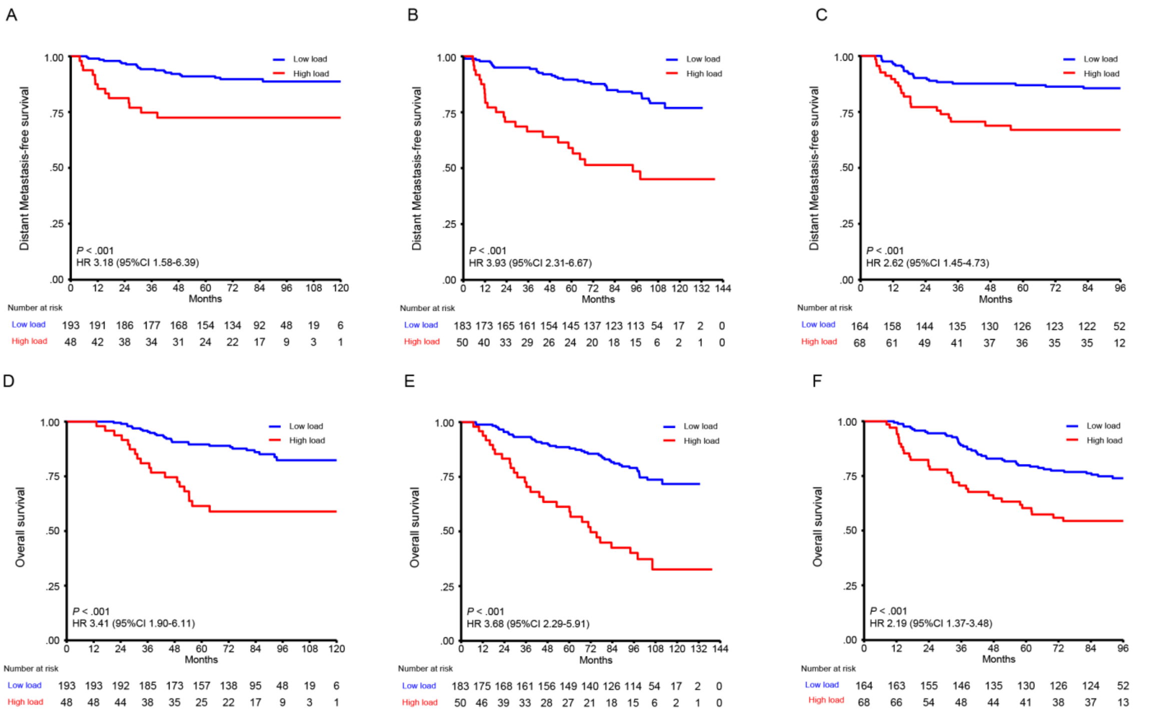

eFigure 14. High intratumoral bacterial load predicts poor prognosis of NPC patients

Kaplan–Meier curves of distant metastasis-free survival and overall survival for the training cohort (n = 241), the internal validation cohort (n = 233) and the external validation cohort (n = 232). We calculated the p value using the unadjusted log-rank test, as well as the hazard ratio and 95% CI using univariable Cox regression analysis. HR indicates hazard ratio.

	Events (n)/ patients (N) in patients with risk factor	Events (n)/ patients (N) in patients without risk factor	HR(95%CI)	P value
**Sex (male vs. female)**				
Training set	55/187	6/54	2.99 (1.29-6.95)	0.01
Internal set	67/167	14/66	2.29 (1.29-4.08)	0.005
External set	61/165	21/67	1.23 (0.75-2.02)	0.41
Overall	183/519	41/187	1.80 (1.28-2.53)	<0.001
**Age (>= 45 years vs. <45 years)**				
Training Set	38/116	23/125	2.04 (1.22-3.43)	0.007
Internal set	47/121	34/112	1.40 (0.90-2.17)	0.14
External set	55/145	27/87	1.27 (0.80-2.02)	0.30
Overall	140/382	84/324	1.57 (1.20-2.06)	0.001
**T stage (T3-4 vs.T1-2)**				
Training set	52/177	9/64	2.29 (1.13-4.64)	0.02
Internal set	61/151	20/82	1.95 (1.18-3.23)	0.01
External set	38/78	44/154	1.99 (1.29-3.08)	0.002
Overall	151/406	73/300	1.68 (1.27-2.23)	<0.001
**N stage (N2-3 vs.N0-1)**				
Training set	38/105	23/136	2.66 (1.58-4.47)	<0.001
Internal set	37/76	44/157	2.22 (1.44-3.45)	<0.001
External set	50/123	32/109	1.52 (0.97-2.37)	0.07
Overall	125/304	99/402	2.02 (1.55-2.63)	<0.001
**TNM stage (III-IV vs. I-II)**				
Training set	58/201	3/40	4.38 (1.37-14.00)	0.01
Internal set	69/175	12/58	2.41 (1.31-4.46)	0.005
External set	71/172	11/60	2.70 (1.43-5.09)	0.002
Overall	198/548	26/158	2.64 (1.75-3.97)	<0.001
**WHO pathological type (Undifferentiated vs. Differentiated)**				
Training set	60/237	1/4	1.15 (0.16-8.33)	0.89
Internal set	77/226	4/7	1.90 (0.70-5.20)	0.21
External set	75/212	7/20	0.94 (0.43-2.03)	0.87
Overall	212/675	12/31	1.32 (0.74-2.36)	0.35
**EBV-DNA (>=2000 vs. <2000)**				
Training set	46/133	15/108	2.88 (1.61-5.16)	<0.001
**Bacterial load (high vs. low)**				
Training set	22/48	39/193	2.90 (1.72-4.90)	<0.001
Internal set	31/50	50/183	3.32 (2.11-5.21)	<0.001
External set	35/68	47/164	2.24 (1.44-3.47)	<0.001
Overall	88/166	136/540	2.76 (2.11-3.61)	<0.001
**Chemotherapy (no vs. yes)**				
Training set	1/18	60/223	5.29 (0.73-38.20)	0.10
Internal set	16/59	65/174	1.59 (0.92-2.76)	0.10
External set	10/30	72/202	1.13 (0.58-2.18)	0.73
Overall	27/107	197/599	1.45 (0.97-2.16)	0.07

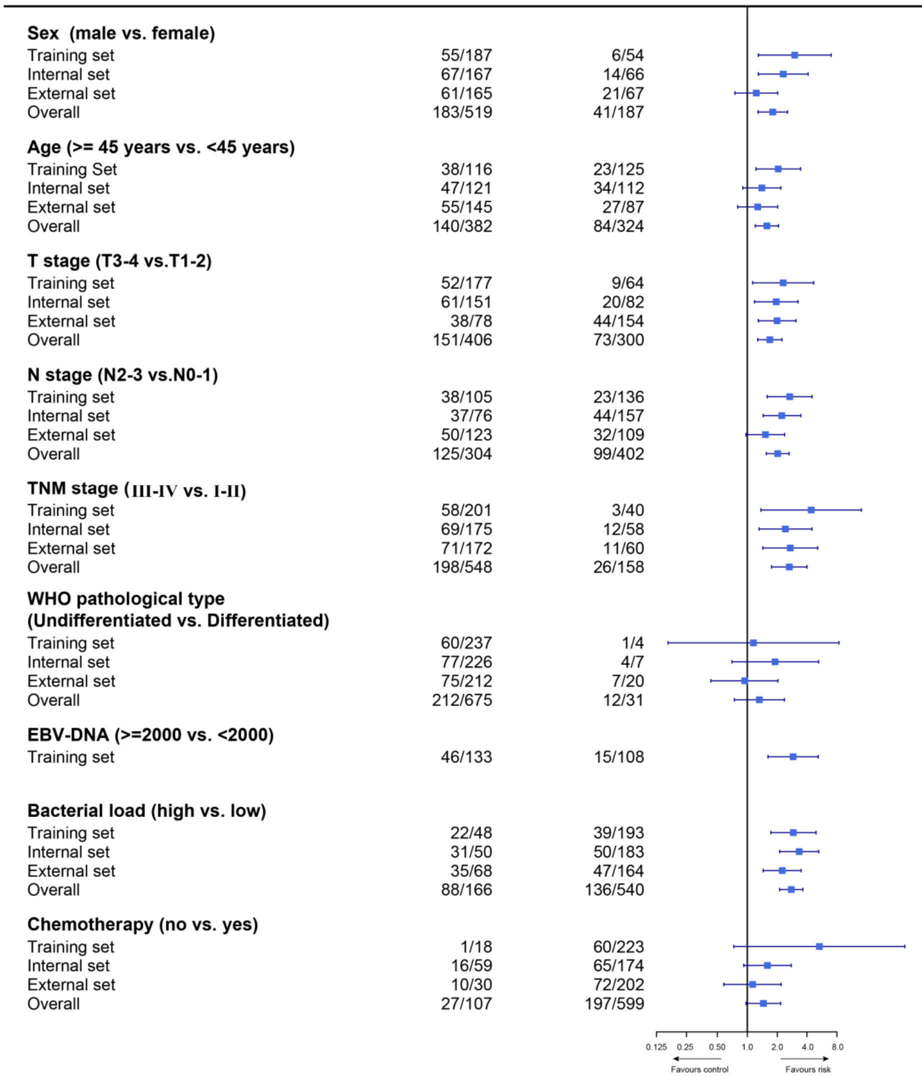

eFigure 15. Univariable Cox analysis of intratumoral bacterial load and clinical characteristics with disease-free survival

HR indicates hazard ratio; EBV, Epstein-Barr virus.

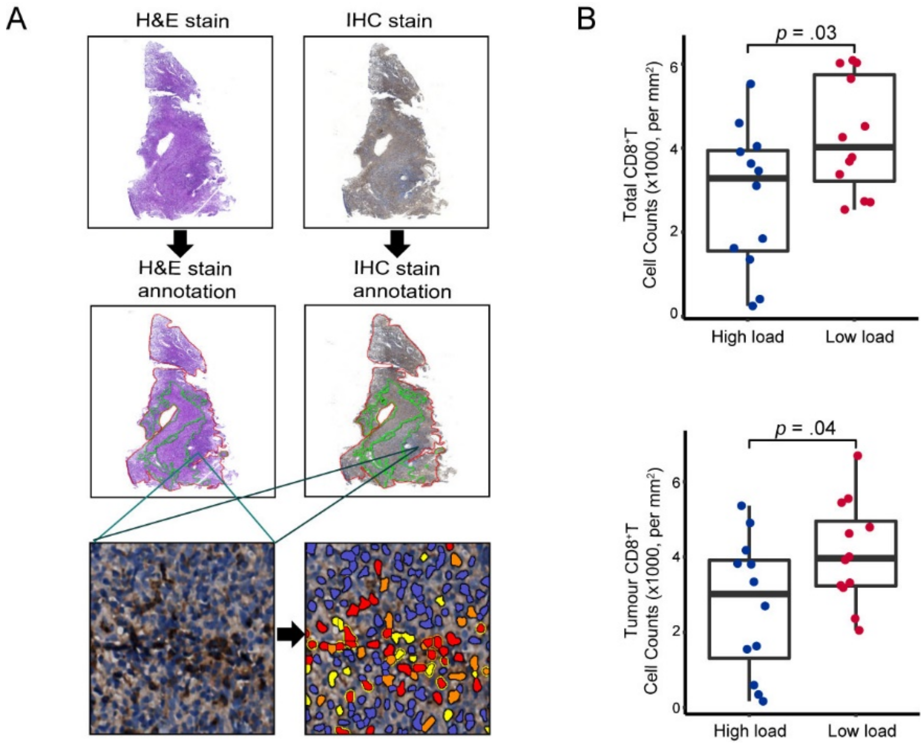

eFigure 16. CD8$^+$ T cell infiltration was validated in 12 pairs of patients with high or low bacterial load

(A) Continuous slides were scanned and the positively stained cells were calculated in HALO software. (B) CD8$^+$ T cell infiltration was validated by immunohistochemistry in tumors with high or low bacterial load. The comparison was performed with paired Wilcoxon signed rank test.

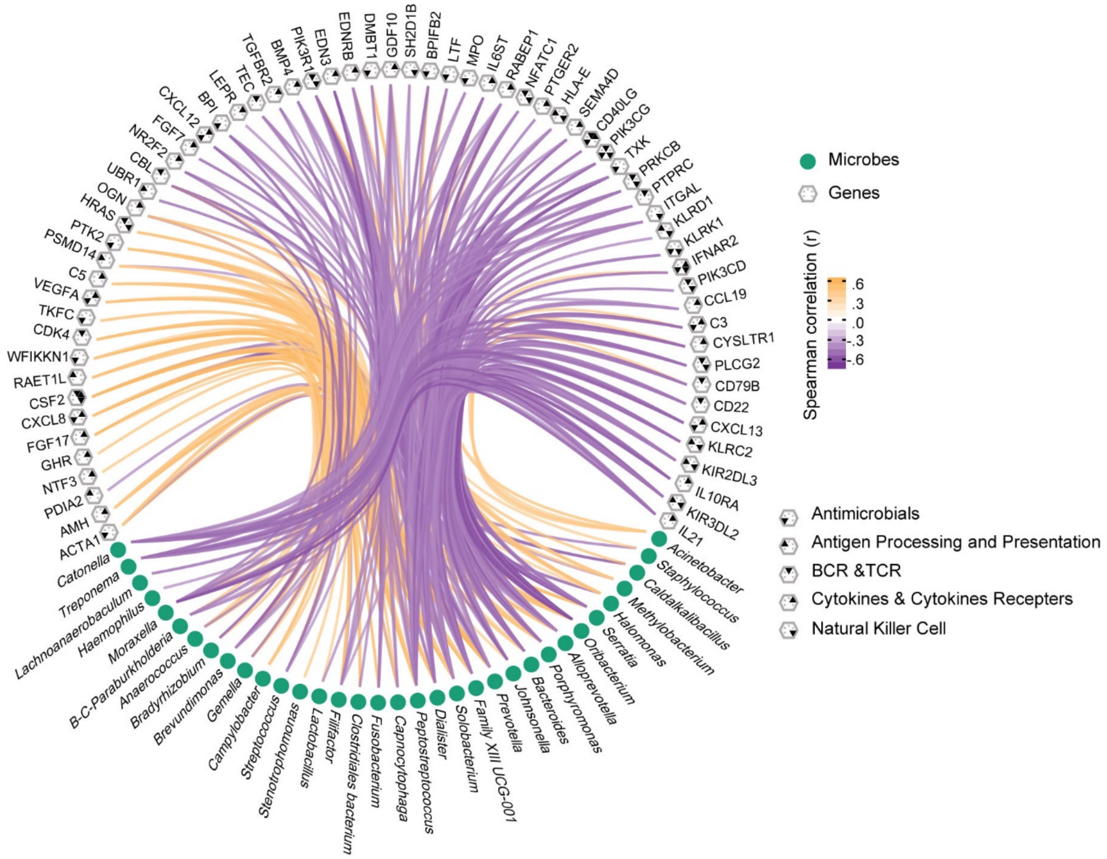

eFigure 17. Network analysis based on the Spearman correlation coefficient of the microbiome and the differentially immunologically expressed genes

Green dots represent the microbes, hexagons represent the target genes (adjusted Benjamini-Hochberg *P* < .05), and black triangles in hexagons represent the immune function in which each gene is involved. The colour of the edge indicates the Spearman correlation of the microbial node and gene node. BCR indicates B-cell receptor; TCR, T- cell receptor.
